# *In Ovo* and dietary administration of oligosaccharides extracted from palm kernel cake influence general health of pre- and neonatal broiler chicks

**DOI:** 10.1371/journal.pone.0184553

**Published:** 2017-09-07

**Authors:** Mohammad Faseleh Jahromi, Parisa Shokryazdan, Zulkifli Idrus, Rohollah Ebrahimi, Juan Boo Liang

**Affiliations:** Institute of Tropical Agriculture, Universiti Putra Malaysia, UPM Serdang, Malaysia; Leibniz-Institut fur Pflanzengenetik und Kulturpflanzenforschung Gatersleben, GERMANY

## Abstract

Palm kernel cake (PKC) is the main byproduct from the palm oil industry in several tropical countries that contains considerable amounts of oligosaccharide. We earlier demonstrated beneficial prebiotic effects of oligosaccharides extract of PKC (OligoPKC) in starter and finisher broiler birds. This study was envisaged to elucidate the effects of *in ovo* and/or oral administration of the OligoPKC on prenatal and post-hatched broiler chicks. A total of 140 broiler (Cobb500) eggs were randomly divided into two groups (n = 70 each), and on day 12 of incubation, eggs in one group received *in ovo* injection of 0.1 mL (containing 20 mg) of OligoPKC, while those in the other group received 0.1 mL of saline (placebo) solution. Of these *in ovo* placebo or OligoPKC injected eggs, after hatching, six chicks from each group were sampled for day-one analysis, while 48 chicks from each group were randomly allocated to two dietary regimes involving either no feeding or feeding of OligoPKC through basal diet for a 14 days experiment forming the experimental groups as: (i) saline-injected (**Control, C**), (ii) OligoPKC-injected (**PREBovo**), (iii) saline-injected, but fed 1% OligoPKC (**PREBd**), and (iv) OligoPKC-injected and also 1% OligoPKC (**PREBovo+d**). *In ovo* injection of prebiotic OligoPKC had no effect on body weight and serum immunoglobulins concentrations of day old chicks, except for IgG, which was increased significantly (P<0.05). Body weight and feed conversion ratio of 14 days old chicks were neither affected by *in ovo* injection nor feeding of OligoPKC. However, populations of cecal total bacteria and major beneficial bacteria of the chicks were markedly enhanced by feeding of OligoPKC (PREBd and PREBovo+d > C and PREBovo), but lesser influenced by *in ovo* OligoPKC injection. Irrespective of its prior *in ovo* exposure, chicks fed OligoPKC diets had lower population of pathogenic bacteria. Overall serum immunoglobulin status of birds was improved by feeding of OligoPKC but *in ovo* OligoPKC injection had minor effect on that. In most cases, *in ovo* OligoPKC injection and feeding of OligoPKC reduced the expression of nutrient transporters in the intestine and improved antioxidant capacity of liver and serum. It is concluded that *in ovo* injection of OligoPKC increased IgG production and antioxidant capacity in serum and liver of prenatal chicks and had limited carrying-over effects on the post-hatched chicks comparing to the supplementary feeding of OligoPKC.

## Introduction

*In ovo* technology has been suggested as a potential alternative for vaccination in broiler chicks. Other applications of this technique in chickens include stimulation of embryogenesis, production of transgenic chickens, and teratogenic effect testing [1]. Dramatic changes in the intestinal weight, villi morphology, and expression and activity of brush-border enzymes and transporters occurrs from day 15 of chicken embryonic development preparing the embryo for exogenous feed ingestion [2]. It thus implies that, intestinal development and maturation during prenatal period has significant influence on growth of newly hatched chicks and thus the long-term effect on broilers performance [2,3].

Although limited information exists, *in ovo* injection of prebiotic has been suggested to potentially improve the balance of intestinal microbiota of chicks after hatching. Pilarski et al. [4] reported that *in ovo* injection of raffinose family oligosaccharides (OS) (RFO) (2.1 mg/egg) and fructo-OS (1.8 mg/egg) at embryonic day 12, significantly increased the population of fecal bifidobacteria of two-day old chicks in comparison with chicks in the sucrose treated and control groups. In addition, the higher level of bifidobacteria population of the prebiotic-injected chicks persisted for the next six weeks. In a large scale broiler experiment (222,400 chickens), Bednarczyk et al. [5] reported that chicks receiving *in ovo* injection of 1.9 mg RFO had similar growth performance compared to their counterparts given antibiotics as growth promoters; however, body weight and FCR were significantly improved for birds receiving prebiotic as compared to those received antibiotics.

Palm kernel cake (PKC) is the main byproduct from the palm oil industry in several tropical countries, including Malaysia, Indonesia, Thailand, and Colombia. Our recent studies showed that PKC contains high level of mannan-oligosaccharides (MOS) with potential prebiotic effects [6,7]. The above investigations also showed that PKC contained about 43% mannose in the form of polymer, and in addition to enhancing the proportion of beneficial to pathogenic microbes, it also enhanced production of immunoglobulins. The aim of this study was to investigate the prebiotic and immune-modulatory effects of *in ovo* administration of MOS extracted from PKC (OligoPKC) on prenatal and neonatal up to 14 days old broiler chicks. Effects of OligoPKC on performance, serum immunoglobulins, intestinal microbiota, nutrient transporters, genes involved in antioxidant capacity of liver and serum in 1 and 14 days old chicks were investigated.

## Materials and methods

### Preparation of OligoPKC

This experiment was conducted in the poultry unit of Institute of Tropical Agriculture, Universiti Putra Malaysia. PKC was provided by a local feed market in Serdang, Selangor, Malaysia. Crude extract of PKC was prepared as follow: 100 g of PKC was dissolved in 1 L of distilled water, shaken for 10 min and autoclaved at 121°C for 20 min. After that, insoluble materials were removed by centrifugation (10000 × g) for 10 min at room temperature (RT), and were filtered through Whatman filter paper No. 1 (Maidstone, UK). To remove the hydrophobic groups, 500 mL of extract was mixed with 400 mL of chloroform/methanol mixture (2:1) [8], shaken for 5 min and kept for 20 min at room temperature. Then, the water/ethanol phase was transferred to another bottle and the same procedure was repeated twice. To remove the proteins, 350 mL of the extract was mixed with 650 mL of acetonitrile, shaken for 5 min and centrifuged at 10000 × g for 10 min at RT, then, the supernatant was separated from the precipitated protein. Water content of the OS extract was removed at 40°C using a Rotary-evaporator (Heidolph Instruments GmbH & Co. KG, Germany) and the residue was freeze dried (FreeZone 6 Liter Benchtop, Labconco Corporation, Kansas City, MO, USA) to obtain the solid OligoPKC.

### Detection of oligosaccharide and monosaccharide using HPLC

Concentrations of OS in the OligoPKC were assessed using HPLC (2690, Waters, USA) with a COSMOSIL Sugar-Dcolumn (250 × 4.6 mm i.d., 5 μm) according to the method described by Jahromi et al. [9]. The mobile phase consisted of acetonitrile and water (65:35 v:v) with flow rate of 0.7 mL/min and column temperature of 35°C. Refractive index detector (2414, Waters, USA) was used for the detection of OS in the sensitivity of 1024 and temperature of 30°C. The sample injection volume was 20 μL, and the running time was 20 min. According to Jaafar and Jarvis [10], the cell wall component of PKC consisted of 580 g/kg mannan, and Zhang et al. [11] reported the extraction of up to 48.8% of D-mannose from PKC, suggesting that major part of OligoPKC is mannan-based. Therefore, five pure MOS, i.e. mannobiose, mannotriose, mannotetraose, mannopentaose and mannohexaose (Megazyme, Ireland) were used as standards for the assay of OS in this study.

Monosaccharides were detected using the same method described for OS, but the mobile phase was changed to 80% acetonitrile in water (instead of 65% for OS) and flow rate was changed to 1 mL/min. glucose, eructose, mannose and xylose (Sigma-Aldrich, St. Louis, MO, USA) were used as standards.

### *In ovo* injection and *in vivo* experiment on chicks

This study was approved by the Ethics Committee of the Universiti Putra Malaysia, and the care and management of chickens and sampling procedures were in compliance with the guidelines of the Federation of Animal Science Societies [12]. Health and welfare of the birds were monitored by a qualified veterinarian who is a member of the research team.

A total of 140 broiler eggs (Cobb500) were obtained from a commercial hatchery (Selangor, Malaysia) and incubated according to standard hatchery practices reported by COBB-VANTRESS, INC, USA (http://www.cobb-vantress.com). *In ovo* injection was conducted according to the method described by Pilarski et al. [4], except that the dosage of prebiotic injection was increased 10 times in comparison to the above report. At 12 days of incubation, the eggs were randomly allocated to two groups of 70 eggs each, and each egg was candled to identify the location of the amnion. The area intended for injection was disinfected with an ethyl alcohol-laden swab (95%). A hole was then punched using a 23-gauge needle and 0.1 mL of OligoPKC solution (containing 20 mg OligoPKC which contained 200 mg/mL OligoPKC in 0.9% saline) or 0.9% saline/placebo (as control) was injected into the amnion to a depth of about 10 mm. The injection area was disinfected again with an ethyl alcohol-laden swab (95%) and sealed with cellophane tape, and the eggs were transferred back to the hatching baskets. After hatching, six chicks from each group (saline- or OligoPKC-injected eggs) were randomly sampled and euthanized by carbon dioxide (99%) inhalation and sacrificed for collection of blood, liver and jejunum samples. Concurrently, 48 chicks from the remaining chicks of each group were randomly selected and allocated to two dietary regimes; namely, basal diet (Table 1) and basal diet supplemented with 1% of OligoPKC (based on our previous study [7]) for a 14 days experiment ultimately constituting the following treatments based on administered prebiotic (PREB), OligoPKC, *in ovo* and/or through diet:

1. Placebo saline-injected **control group (C)**
2. **OligoPKC-injected (PREBovo)**
3. Placebo saline-injected and **fed 1% OligoPKC (PREBd)**
4. **OligoPKC-injected and fed 1% OligoPKC (PREBovo+d)**

**Table 1:** Composition and nutrient contents of basal diet.

Ingredient	(g/kg unless otherwise stated)
Ground yellow corn	538.9
Soybean meal (CP 40 to 42%)	361.9
Fish meal (CP 70%)	30.0
Palm oil	37.4
Choline chloride (60%)	2.5
Trimix^1^	1.0
Salt (NaCl)	2.0
DL-methionine (99%)	1.8
Limestone	13.0
Dicalcium phosphate	11.5
Total	1000.0
Calculated nutrients content	
Crude protein	220.0
Crude fat	63.1
Crude fiber	38.0
Calcium	10.2
Phosphorus	4.5
Metabolisable energy (MJ/kg)	13.06

^1^Trimix (per kg Trimix): iron 100 g; manganese 110 g; copper 20 g; zinc 100 g; iodine 2 g; selenite 0.2 g; cobalt 0.6 g; santoquin 0.6 g; folic acid 0.33 g; thiamin 0.83 g; pyridoxine 1.33 g; biotin 2% 0.03 g; riboflavin 2 g; cyanocobalamin 0.03 g; D-calcium pantothenate 3.75 g; niacin 23.3 g; retinol 2000 mg; cholecalciferol 25 mg; α-tocopherol 23,000 mg IU.

Chicks were weighted on day one and chicks from each treatment groups were randomly allocated into 6 replicate cages (4 chicks/ pen). The cages (90 × 56 × 50 cm) were located in an open-sided poultry experimental house exposed to 23 h lighting and 1 h dark (from 21:00–22:00). The chicks had *ad libitum* access to feed and clean drinking water. On day 14, birds were euthanized by carbon dioxide (99%) inhalation followed by sacrificing, and cecal contents were collected to investigate the microbial population. Concurrently, blood samples were collected for determination of the immunoglobulins concentrations and antioxidant capacity of serum. Liver and jejunum samples were also collected for antioxidant capacity and genes expression studies.

### Determination of immunoglobulins concentrations in the serum

The concentrations of immunoglobulins A, G and M (IgA, IgG and IgM) in the serum were measured using commercial ELISA kit for chickens (Cusabio Biotech Co., Ltd., Wuhan, China) according to the manufacturer’s instructions.

### DNA extraction and microbial quantification of cecal contents

Microbial quantification of cecal contents was performed according to the method described by Jahromi et al. [9]. Total DNA was extracted from cecal samples and pure cultures of target bacteria (listed in Table 2) using the QIAamp DNA Stool Mini Kit (Qiagen Inc., Valencia, CA) according to the manufacturer’s instructions. The extracted DNA was stored at -20°C until use. The extracted DNA from pure cultures was used for production of high concentrations of target DNA using the normal PCR and preparation of the standard curves. The PCR products were purified using the MEGA quick-spin^TM^ kit (Intron Biotechnology, Inc, South Korea) and the purity and concentration of DNA in each sample were measured using a Nanodrop ND-1000 spectrophotometer (Thermo Scientific, USA). The number of copies of each template DNA per mL of elution buffer was calculated using the following formula that is available online (http://cels.uri.edu/gsc/cndna.html):

$$\mathrm{Number}\;\mathrm{of}\;\mathrm{copies}=\frac{\mathrm{Amount}\;\mathrm{of}\;\mathrm{DNA}\;(\mathrm{\mu g}/\mathrm{mL})\times 6.022\times 10^{23}}{\mathrm{Length}\;(\mathrm{bp})\times 10^{9}\times 650}$$

**Table 2:** Primer used in bacterial quantification.

Microorganism	Primer	Size of amplified fragments (bp)	Annealing temperature (°C)
Total bacteria	F-5′-CGGCAACGAGCGCAACCC-3′ R-5′-CCATTGTAGCACGTGTGTAGCC-3′	145	55
*Lactobacillus*	F-5′-CATCCAGTGCAAACCTAAGAG-3′ R-5′-GATCCGCTTGCCTTCGCA-3′	341	58
E*nterococcus*	F-5′-CCCTTATTGTTAGTTGCCATCATT-3′ R-5′-ACTCGTTGTACTTCCCATTGT-3′	144	50
*Bifidobacterium*	F-5′- GGGTGGTAATGCCGGATG-3′ R-5′- TAAGCCATGGACTTTCACACC-3′	440	60
E*scherichia coli*	F-5′-GTGTGATATCTACCCGCTTCGC-3′ R-5′-AGAACGCTTTGTGGTTAATCAGGA-3′	82	50
*Enterobacteriace*	F- 5′-CAT TGACGTTACCCGCAGAAGAAGC-3′ R-5′-CTCTACGAGACTCAAGCTTGC-3′	195	50
*Salmonella*	5’-TCGTCATTCCATTACCTACC-3’ 5’-AAACGTTGAAAAACTGAGGA-3’	119	50
Clostridiaceae	F-5′-GAG TTT GAT CMT GGC TCA G-3′ R-5′- CCC TTT ACA CCC AGT AA-3′	552	55
*Campylobacter*	F: 5-GGATGACACTTTTCGGAG-3 R: 5-AATTCCATCTGCCTCTCC-3	246	55

For each standard curve, the number of copies of 16S rRNA gene was plotted against cycle threshold (CT) values obtained from real-time PCR. Real-time PCR reactions were performed with a BioRadCFX96 Touch machine (BioRad, USA) using optical grade plates. Each reaction was performed on a total volume of 25 μL using the iQTMSYBR Green Supermix (BioRad, BioRad, USA). Each reaction included 12.5 μL SYBR Green Supermix, 1 μL of each forward primer (Table 2), 1 μL of each reverse primer (Table 2), 1 μL of DNA sample and 9.5 μL nuclease-free water. The reaction conditions for amplification of DNA were 94°C for 5 min, followed by 40 cycles of denaturation at 94°C for 20 s; annealing (according to Table 2) for 30 s; and extension at 72°C for 20 s. To confirm the specificity of amplification, melting curve analysis was carried out after the last cycle of each amplification. The expected sizes of amplified fragments are presented in Table 2. The estimated values were expressed as bacterial cell number per gram cecal contents.

### Gene expression analysis

Gene expression analysis was conducted according to the method described previously [13]. Antioxidant genes were analyzed in the liver and nutrient transporter genes were analyzed in the intestine. Samples of the liver and intestine were removed, immediately flushed with ice-cold saline, frozen in liquid nitrogen, and stored at -80°C until use. Total RNA was extracted from the liver samples (6 samples per treatment) using the RNeasy Midi Kit (Qiagen Inc., Valencia, CA) according to the manufacturer’s instructions. Total RNA was quantified at 260/280 nm using a NanoDropND-1000 spectrophotometer (Thermo Scientific, Wilmington, DE, USA) and stored at -80°C until use. The quality and integrity of RNA was checked through agarose gel electrophoresis. Two μg of total RNA was reverse-transcribed using cDNA synthesis Kit according to the manufacturer’s instructions (Maxime RT-PCR Kit, iNtRON, Seongnam, Korea).

The isolated RNA was subjected to quantitative real-time PCR using the SYBR green assay with SYBR green PCR master mix (Thermo Scientific, Wilmington, DE, USA), and using a Biorad CFX96™ real-time cycler (Bio-Rad Laboratories Inc, Hercules, USA). Each PCR reaction was performed on a total volume of 25μL using SYBR green PCR master mix (Thermo Scientific, Wilmington, DE, USA). Each reaction included 12.5μL SYBR Green, 1 μL of each forward primer (Table 3), 1 μL of each reverse primer (Table 3), 1μL of cDNA samples, and 9.5μL nuclease-free water. Glyceraldehyde phosphate dehydrogenase (GAPDH) and β-actin were used as reference genes.

**Table 3:** Primer sequences (5′→3′) used in the gene expression procedure.

Name	Forward primer	Reverse primer	Annealing temperature	Product size (bp)	GenBank ID	Reference
SGLT1	TGTCTCTCTGGCAAGAACATGTC	GGGCAAGAGCTTCAGGTATCC	60	229	XM_415247	Gilbert et al., [35]
SGLT5	ATACCCAAGGTAATAGTCCCAAAC	TGGGTCCCTGAACAAATGAAA	60	75	XM_422459	Gilbert et al., [35]
GLUT2	CACACTATGGGCGCATGCT	ATTGTCCCTGGAGGTGTTGGTG	60	116	Z22932	Gilbert et al., [35]
GLUT5	TTGCTGGCTTTGGGTTGTG	GGAGGTTGAGGGCCAAAGTC	60	99	XM_417596	Gilbert et al., [35]
PepT1	CCCCTGAGGAGGATCACTGTT	CAAAAGAGCAGCAGCAACGA	60	205	NM_204365	Gilbert et al., [35]
EAAT3	TGCTGCTTTGGATTCCAGTGT	AGCAATGACTGTAGTGCAGAAGTAATATATG	60	79	XM_424930	Gilbert et al., [35]
LCAD	CAGTGACTTGCAAGGAGTACGGA	AGCCTCTCGGTTTGTAACCGTAA	65	136	XM_421861	Ebrahimi et al [13]
CAT	GGGGAGCTGTTTACTGCAAG	TTTCCATTGGCTATGGCATT	60	139	AJ719360.1	Ebrahimi et al [13]
SOD	AGGGGGTCATCCACTTCC	CCCATTTGTGTTGTCTCCAA	62	122	NM_205064.1	Ebrahimi et al [13]
GST-α	GCCTGACTTCAGTCCTTGGT	CCACCGAATTGACTCCATCT	62	131	L15386.1	Ebrahimi et al [13]
GAPDH	GCCGTCCTCTCTGGCAAAG	TGTAAACCATGTAGTTCAGATCGATGA	60	128	NM204305	Ebrahimi et al [13]
β-actin	ATGAAGCCCAGAGCAAAAGA	GGGGTGTTGAAGGTCTCAAA	60	175	X00182	Ebrahimi et al [13]

SGLT1, Na+-dependent glucose and galactose transporter; SGLT5, glucose transporter;GLUT2, Na+-independent glucose, galactose and fructose transporter;GLUT5, Na+-independent fructose transporter;PepT1, oligopeptide transporter; EAAT3, excitatory amino acid transporter; LCAD, long-chain acyl CoA dehydrogenase; CAT, catalase; SOD, superoxide dismutase; GST-α, glutathione S-transferase-α; GAPDH, glyceraldehyde phosphate dehydrogenase.

PCR process was performed under the following conditions: 95°C for 2 min, 40 cycles of 95°C for 20 s, annealing temperature (according to Table 3) for 30s and 72°C for 20s. To confirm the specificity of amplification, melting curve analysis was carried out after the last cycle of each amplification by increasing the temperature from 60°C to 95°C (continues increasing of 0.5°C and hold for 5s). The cycle numbers at which amplified DNA samples exceeded a computer-generated fluorescence threshold level were normalized and compared with determined relative gene expression. Higher cycle number values indicated lower initial concentrations of cDNA and thus, lower levels of mRNA expression. The ΔΔCT method was used for determination of relative gene expression [14], where ΔCT is difference in CT value of the target gene from the CT value of the reference gene (GAPDH or β-actin). The ΔΔCT is ΔCT of treated samples minus ΔCT of the control. Data are presented as fold change expression in the target gene of a treated sample compared to the control samples.

### Antioxidant activity of the liver and serum

The 2,2'-azino-bis (3-ethylbenzothiazoline-6-sulfonic acid) (ABTS) and ferric reducing ability of plasma (FRAP) assays were used for determination of antioxidant capacity of the liver and serum samples. The amount of 200 mg of each liver sample was homogenized in 1 mL of cold phosphate buffer saline (PBS) (8 g NaCl, 0.2 g KCl, 1.44 g Na_2_HPO_4_, 0.24 g KH_2_PO_4_ in 1 L distilled water, pH 7.2) on ice, and then centrifuged at 10000 × g for 15 min at 4°C. After that the supernatant was kept at -80°C until use. Similarly, 1 mL of each serum sample was centrifuged at 10000 × g for 15 min at 4°C and the supernatant was kept at -80°C until use.

The ABTS assay followed the method described by Tsai *et al*. [15] with minor modifications. The ABTS stock solutions were 7.4 mM ABTS and 2.6 mM potassium persulfate. The ABTS radical cation (ABTS°^+^) was prepared by mixing the two stock solutions in equal quantities (1:1, v:v) and allowing them to react for 12 h at room temperature in the dark, after which the mixture was diluted by adding methanol to obtain an absorbance of 1.1 ± 02 at 734 nm using a spectrophotometer (Barnstead International, USA). After that, 200 μL of ABTS°^+^ solution was added to 50 μL of each sample. The mixtures were incubated for 5 min at room temperature in the dark, and absorbance was determined at 734 nm. Different concentrations of Trolox (25 to 1000 μmol/L) were used to prepare the standard curve, and the results were expressed as Trolox Equivalents Antioxidant Capacity (TEAC) in the form of μmol Trolox/g liver or mL serum.

The FRAP assay was carried out according to the method developed by Benzie and Strain [16] with minor modifications. The FRAP reagent was prepared by mixing 300 mM acetate buffer (pH 3.6, containing 6.4 mL 2 M sodium acetate and 93.6 mL 2 M acetic acid), 10 mM 2,4,6-tri (2-pyridyl)-1,3,5-triazine (TPTZ) in 40 mM HCl, and 20 mM ferric chloride in the ratio of 10:1:1 (v:v:v). After that, 200 μL of prepared FRAP reagent was added to 50 μL of each sample. The mixture was incubated for 30 min at room temperature in the dark, after which the absorbance was determined at 593 nm using a spectrophotometer (Barnstead International, USA). The FRAP reagent was used as blank and the final absorbance of each sample was compared with those obtained from the standard curve, made from 0 to 1000 μmol/L ferric sulphate (FeSO4-7H2O). The results were expressed in μmol Fe2^+^/g liver or mL serum.

A summary of experimental design is presented in S1 Appendix.

### Statistical analysis

All data analysis of *in ovo* and *in vivo* experiments was conducted using six replicates. All data were analyzed by one-way ANOVA procedure of SAS program [17] version 9.2., except for the results of experiments with only two treatments (data from day-old chicks) which T-test was used. Results were expressed as means ± SD. Alpha level of 0.05 was used as the critical level of significance.

## Results

Hatchability was numerically higher for the OligoPKC-injected eggs (Table 4). However, *in ovo* injection of OligoPKC had no effect on weight of 1-day old chicks, growth parameters, feed intake and FCR of birds at day 14 of age (P>0.05) (Table 5).

**Table 4:** Effect of *in ovo* injection of prebiotic, oligosaccharides extract of palm kernel cake (OligoPKC), on hatchability and weight of one-day-old chicks (n = 70).

Treatments	Hatchability (%)	Chicks weight (g)	
		Mean	Range
Control (Saline injection)	89.83	44.11±3.17	35.66–51.62
OligoPKC injection	95.52	43.63±2.98	36.80–51.72

Values are means ± SD.

**Table 5:** Effect of prebiotic, oligosaccharides extract of palm kernel cake (OligoPKC), injected *in ovo* and/or fed through diet on performance of broiler chicks during one to 14 days of age.

Parameter	Treatment			
	C	PREBovo	PREBd	PREBovo+d
Initial weight (g)	43.93±0.86	44.38±1.59	44.87±1.09	43.97±1.72
Final weight (g)	417.90±17.98	421.80±15.75	424.05±14.84	419.10±13.31
Body weight gain (g)	373.97±18.29	377.42±14.42	379.18±15.22	375.13±12.25
Feed intake (g)	503.94±15.11	497.84±3.44	510.05±6.32	493.03±3.44
FCR (g/g)	1.349±0.057	1.321±0.055	1.347±0.070	1.315±0.044

Values are means ± SD of 6 replicates. C, control; PREBovo, 20mg OligoPKC *in ovo*-injected; PREBd, fed 1% OligoPKC; PREBovo+d, 20 mg OligoPKC *in ovo*-injected + fed 1% OligoPKC. Parallel to *in ovo* injection of OligoPKC, an active component, saline as placebo was injected.

Among the three serum immunoglobulins (IgA, IgM and IgG) determined for 1-day old chicks, only IgG was significantly enhanced by *in ovo* OligoPKC injection (P<0.05) compared to the control (Table 6). However, *in ovo* OligoPKC injection had no influence on the concentration of the three immunoglobulins in serum of 14 days old chicks compared to the control (P>0.05). On the other hand, feeding of OligoPKC (PREBd group) for 14 days post-hatching significantly increased (P<0.05) the values of these three serum immunoglobulins. Prior *in ovo* exposure of OligoPKC didn't further potentiate its effect when given in diet as evidenced by similar values of these immunoglobulins in chicks of PREBd and PREBovo+d groups. No significant effect was observed in the tested biochemical parameters of serum by receiving OligoPKC (Table 7).

**Table 6:** Effect of prebiotic, oligosaccharides extract of palm kernel cake (OligoPKC), on serum immunoglobulins concentrations of chicks at one and 14 days of age.

	IgA (μg/mL)	IgM (μg/mL)	IgG (mg/mL)
1 day of age			
Control (Saline injection)	2506±273	303±72	5.83±0.64^b^
OligoPKC injection	2709±219	335±53	8.50±0.80^a^
14 days of age			
C	189±42^b^	303±63^b^	5.49±0.83^c^
PREBovo	199±60^b^	357±34^a^^b^	5.61±0.48^b^^c^
PREBd	325±76^a^	392±51^a^	6.32±0.81^a^^b^
PREBovo+d	330±44^a^	399±66^a^	6.95±0.56^a^

Values are means ± SD of 6 replicates.

^a-c^ means in each column with different superscripts differ significantly (P<0.05). C, control; PREBovo, 20mg OligoPKC *in ovo*-injected; PREBd, fed 1% OligoPKC; PREBovo+d, 20 mg OligoPKC *in ovo*-injected + fed 1% OligoPKC. Parallel to *in ovo* injection of OligoPKC, an active component, saline as placebo was injected.

**Table 7:** Effect of prebiotic, oligosaccharides extract of palm kernel cake (OligoPKC), on biochemical serum parameters at day 14 of age.

Parameter		Treatment		
	C	PREBovo	PREBd	PREBovo+d
urea (mmol/L)	1.30±0.39	1.25±0.13	1.40±0.32	1.20±0.08
Uric acid (mmol/L)	0.31±0.04	0.39±0.20	0.32±0.11	0.25±0.11
Calcium (mmol/L)	2.30±0.13	2.46±0.15	2.34±0.25	2.39±0.12
Corrected calcium (mmol/L)	2.87±0.11	3.03±0.14	2.90±0.22	2.97±0.10
Total protein (g/L)	25.00±4.24	25.00±2.16	24.75±2.22	24.25±2.50
Albumin (g/L)	11.50±1.73	11.75±1.26	11.75±1.71	11.25±1.50
Globulin (g/L)	13.50±2.52	13.25±0.96	13.00±0.82	13.00±1.83
Albumin /globulin ratio	0.83±0.05	0.88±0.05	0.90±0.12	0.88±0.13
Total cholesterol (mmol/L)	2.33±0.26	2.35±0.26	2.33±0.33	2.20±0.20
Triacylglycerol (mmol/L)	0.68±0.18	0.62±0.09	0.70±0.12	0.69±0.09
HDL (mmol/L)	1.70±0.27	1.65±0.09	1.75±0.25	1.55±0.15
LDL (mmol/L)	0.32±0.02	0.42±0.15	0.26±0.15	0.33±0.21
Total cholesterol/HDL ratio	1.38±0.10	1.43±0.10	1.33±0.10	1.43±0.15
glucose (mmol/L)	11.75±1.12	10.55±1.32	12.50±1.92	11.85±1.76

Values are means ± SD of 6 replicates. C, control; PREBovo, 20mg OligoPKC *in ovo*-injected; PREBd, fed 1% OligoPKC; PREBovo+d, 20 mg OligoPKC *in ovo*-injected + fed 1% OligoPKC. Parallel to *in ovo* injection of OligoPKC, an active component, saline as placebo was injected.

When comparing to the control (C) group, *in ovo* injection of OligoPKC (PREBovo) did not cause any significant difference in the populations of gut microorganisms at day 14 of age, except for the *Bifidobacterium* genus which was increased. However, feeding of OligoPKC (PREBd and PREBovo+d) significantly altered (P<0.05) the population of gut microorganisms, except for the *Enterococcus* genus (Table 8). There was no significant difference between PREBd (receiving OligoPKC through diet) and PREBovo+d (receiving OligoPKC through both *in ovo*-injection and diet) groups in terms of their effects on gut microbes. Irrespective of given *in ovo* injection or not, populations of total bacteria and beneficial bacteria (*Lactobacillus* and *Bifidobacterium*) in OligoPKC-fed birds (PREBovo+d and PREBd) were higher (P<0.05) than those not receiving the same dietary treatment (C and PREBovo), except that no difference (P>0.05) was observed for total microbes and *Lactobacillus* between the PREBd and PREBovo groups. The latter together with the higher population of *Bifidobacterium* in the PREBovo compared to the control (C) seems to suggest a beneficial carryover effect of *in ovo* OligoPKC treatment in enhancing gut microbiota in chicks. However, irrespective of OligoPKC injection, almost in all cases, chicks received dietary supplementation of OligoPKC had lower population of pathogenic bacteria (P<0.05) as compared to those not receiving the same dietary treatment.

**Table 8:** Effect of prebiotic, oligosaccharides extract of palm kernel cake (OligoPKC), on intestinal microbial population (Log^10^ copy number/g) at day 14 of age.

Bacteria		Treatment		
	C	PREBovo	PREBd	PREBovo+d
Total microbes	9.74±0.24^c^	9.96±0.14^b^^c^	10.17±0.15^a^^b^	10.36±0.14^a^
*Lactobacillus*	6.57±0.15^c^	6.82±0.23^b^^c^	7.30±0.18^a^^b^	7.67±0.34^a^
*Bifidobacterium*	6.22±0.26^c^	6.72±0.22^b^	7.24±0.08^a^	7.37±0.07^a^
*Enterococcus*	6.57±0.11	6.67±0.18	6.78±0.32	6.74±0.24
*Escherichia coli*	6.55±0.27^a^	6.25±0.23^a^	5.89±0.21^b^	5.67±0.15^b^
*Enterobacteriace*	7.17±0.31^a^	6.92±0.19^a^^b^	6.62±0.16^b^	6.51±0.32^b^
Clostridiaceae	6.37±0.22^a^	6.05±0.08^a^	5.10±0.19^b^	5.18±0.38^b^
*Salmonella*	3.17±0.33^a^	3.10±0.38^a^	2.55±0.22^b^	2.38±0.19^b^
*Campylobacter*	6.79±0.18^a^	6.46±0.39^a^^b^	6.03±0.19^b^	6.08±0.24^b^

Values are means ± SD of 6 replicates.

^a-c^ means in each row with different superscripts differ significantly (P<0.05). C, control; PREBovo, 20mg OligoPKC *in ovo*-injected; PREBd, fed 1% OligoPKC; PREBovo+d, 20 mg OligoPKC *in ovo*-injected + fed 1% OligoPKC. Parallel to *in ovo* injection of OligoPKC, an active component, saline as placebo was injected.

**Fig 1:**
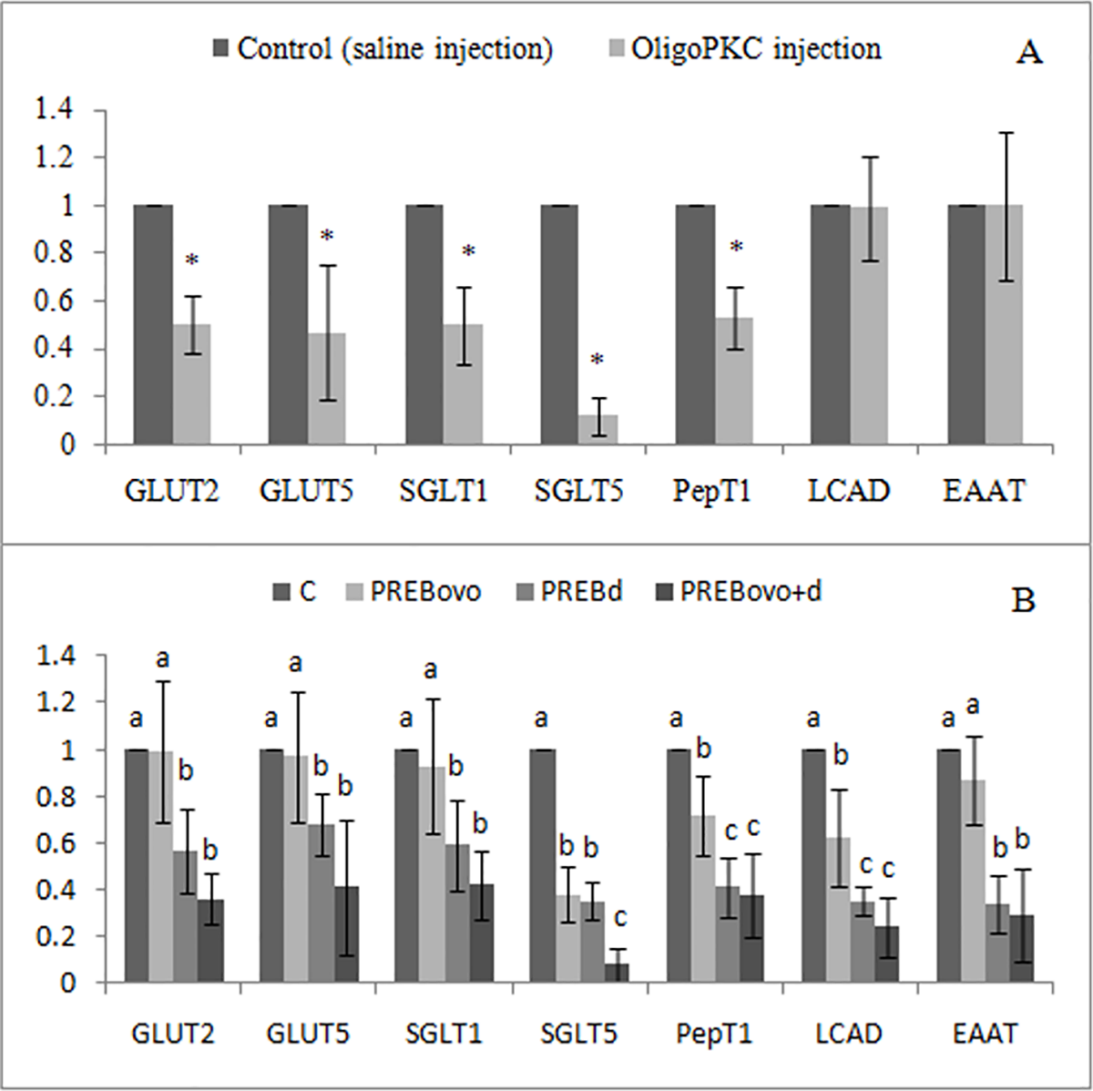
Effect of prebiotic, oligosaccharides extract of palm kernel cake (OligoPKC), on expression of nutrient transporter genes of jejunum. A: 1 day of age; B: 14 days of age. Values are expressed as means of six replicates, Error bars are standard deviation. a–c, bars with different letters are significantly different (P<0.05). * indicates P<0.05 by T-test. C, control; PREBovo, 20mg OligoPKC *in ovo*-injected; PREBd, fed 1% OligoPKC; PREBovo+d, 20 mg OligoPKC *in ovo*-injected + fed 1% OligoPKC; SGLT1, Na+-dependent glucose and galactose transporter; SGLT5, glucose transporter; GLUT2, Na+-independent glucose, galactose and fructose transporter; GLUT5, Na+-independent fructose transporter; PepT1, oligopeptide transporter; EAAT3, excitatory amino acid transporter; LCAD, Long-chain acyl CoA dehydrogenase. Parallel to *in ovo* injection of OligoPKC, an active component, saline as placebo was injected.

The effects of OligoPKC on expression of nutrient transporter genes are presented in Fig 1. For the 1-day-old chicks, *in ovo* injection of OligoPKC significantly (P<0.05) down-regulated the expression of the four intestinal sugar transporter genes (GLUT2, GLUT5, SGLT1 and SGLT5) and peptide transporter PepT1, but not those of LCAD and amino acid transporter EAAT. As for the 14-day-old chicks, chicks received *in ovo* OligoPKC injection and no OligoPKC in diet (PREBovo) had lower expression of SGLT5, PepT1 and LCAD genes than those in the control group (C). Both groups of birds receiving dietary OligoPKC supplementation (PREBd and PREBovo+d) showed lower expressions of all the nutrient transporter genes than those received OligoPKC only through *in ovo* injection (PREBovo) and the control group, except for SGLT5 which in there was no significant difference between PREBovo and PREBd groups.

**Fig 2:**
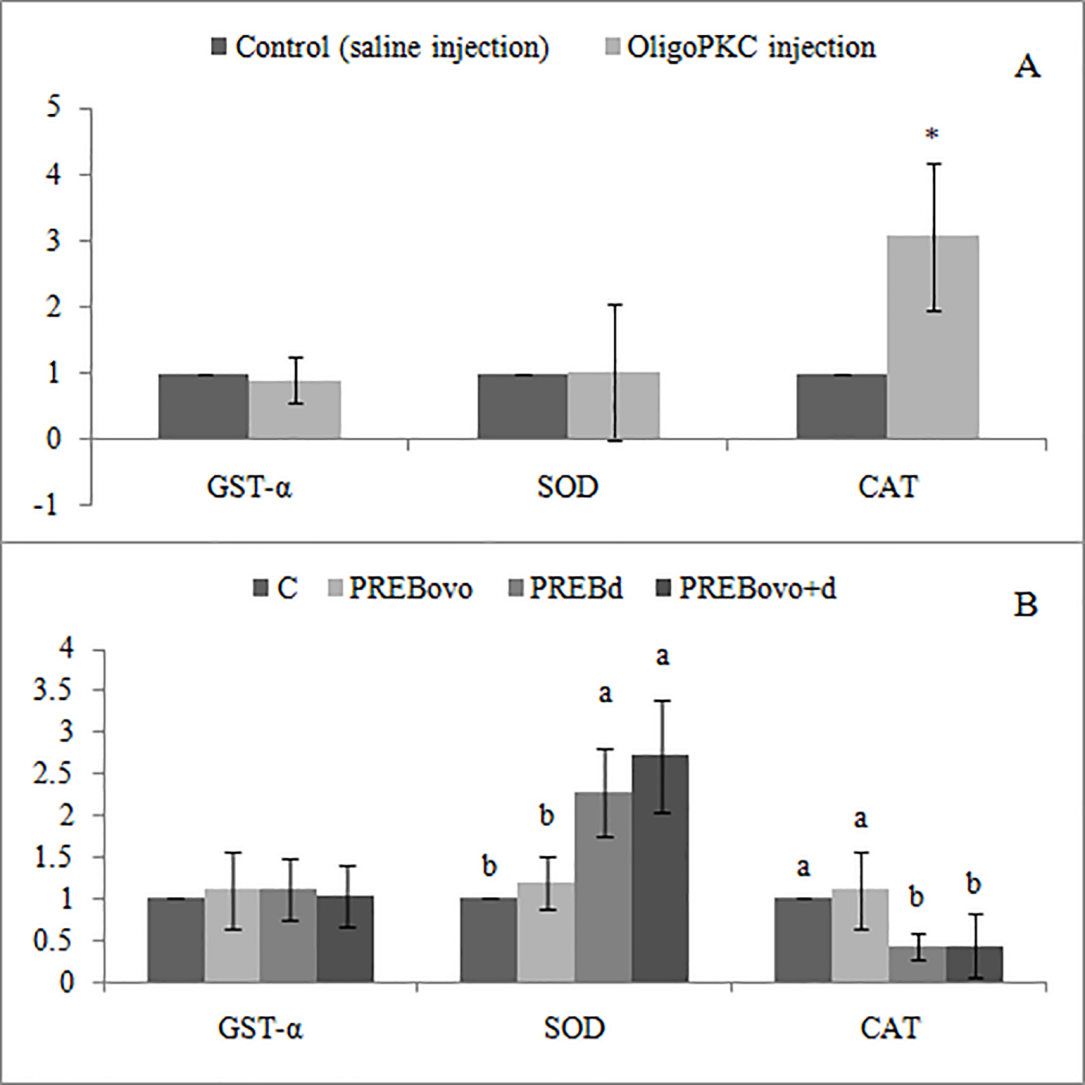
Effect of prebiotic, oligosaccharides extract of palm kernel cake (OligoPKC), on expression of genes involve in production of antioxidant enzymes in the liver. A: 1 day of age; B: 14 days of age. Values are expressed as means of six replicates. Error bars are standard deviation. a–b, bars with different letters are significantly different (P<0.05). * indicates P<0.05 by T-test. C, control; PREBovo, 20mg OligoPKC *in ovo*-injected; PREBd, fed 1% OligoPKC; PREBovo+d, 20 mg OligoPKC *in ovo*-injected + fed 1% OligoPKC; CAT, catalase; SOD, superoxide dismutase; GST-α, glutathione S-transferase-α. Parallel to *in ovo* injection of OligoPKC, an active component, saline as placebo was injected.

As regard to the antioxidant related genes (antioxidant enzymes), at first day post-hatching, expression of catalase (CAT) in the liver of one-day old chicks received *in ovo* OligoPKC-injection was significantly higher (P< 0.05) than that of the control group. However, no changes were observed in the expression of glutathione S-transferase-α (GST-α) and superoxide dismutase (SOD) (Fig 2A).

At day 14 of age (Fig 2B), birds receiving *in ovo* injection of OligoPKC in comparison to the control group did not showed any significant difference in expression of genes involved in antioxidant capacity. However, dietary supplementation of OligoPKC (PREBd and PREBovo+d) significantly (P<0.05) up-regulated the expression of SOD, and down-regulated the expression of CAT in comparison to chicks fed no OligoPKC (C and PREBovo). There was no significant difference in the expression of GST-α between the four treatment groups. In addition, no significant difference was detected between PREBd and PREBovo+d groups in expression of the three antioxidant genes.

Total antioxidant capacities of serum and liver of one-day-old chicks were significantly (P<0.05) higher in the OligoPKC-injected group than the control group, measured by both FRAP and ABTS methods (Table 9). At 14 days of age, *in ovo* injection of OligoPKC in PREBovo group caused no significant difference in the total antioxidant capacities of serum and liver when comparing to the control group (P>0.05). However, total antioxidant capacities of serum and liver were significantly higher (P<0.05) in chicks fed with OligoPKC supplemented diets (PREBd and PREBovo+d) in compression to those of the control group (C). There was no significant difference between PREBd and PREBovo+d groups.

**Table 9:** Effect of prebiotic, oligosaccharides extract of palm kernel cake (OligoPKC), on antioxidant capacities of serum and liver at one and 14 days of age.

	FRAP (μmol Fe2^+^/mL or g)		ABTS (μmol Trolox/mL or g)	
	serum	liver	serum	liver
1 day of age				
Control	5.52±0.63^b^	7.62±0.70^b^	7.47±0.31^b^	10.72±0.8^b^
OligoPKC-injected	9.86±1.04^a^	10.34±0.94^a^	9.86±0.60^a^	13.17±0.78^a^
14 days of age				
C	6.35±0.58^b^	9.95±1.10^b^	8.21±0.64^b^	11.62±0.85^b^
PREBovo	6.70±0.89^b^	10.53±0.54^a^^b^	9.05±1.24^a^^b^	11.70±1.11^b^
PREBd	8.43±1.56^a^	11.33±0.56^a^	9.42±1.03^a^	13.59±0.86^a^
PREBovo+d	8.36±0.62^a^	11.45±1.28^a^	9.85±0.55^a^	13.65±1.04^a^

Values are means ± SD of 6 replicates.

^a-b^ for first day or 14 days of age, means in each column with different superscripts differ significantly (P<0.05). C, control; PREBovo, 20mg OligoPKC *in ovo*-injected; PREBd, fed 1% OligoPKC; PREBovo+d, 20 mg OligoPKC *in ovo*-injected + fed 1% OligoPKC. Parallel to *in ovo* injection of OligoPKC, an active component, saline as placebo was injected.

## Discussion

We have previously reported that the beneficial effects of OligoPKC in animals are mainly because of its prebiotic MOS contents that increase beneficial gut microbiota and immunoglobulins production [6]. In the present study, *in ovo* injection and feed supplementation of OligoPKC had no significant effect on the performance of broiler chicks reared until 14 days. However, in previous study though significant positive effect of OligoPKC on FCR at 42 days of age was observed [7]. In a 42 days experiment on broiler chickens, Bednarczyk et al. [5] reported that *in ovo* injection of RFO significantly increased body weight and FCR. On the other hand, Pilarski et al. [4] reported no effect on growth performance of chicks given *in ovo* RFO.

Although vaccination, antibiotic supplementation and hygienic environmental management are common methods used to control diseases and improving the productivity of poultry, improvement of the immunity of newly hatched chicks using *in ovo* injection of prebiotic has been suggested as an alternative option to the above-mentioned strategies [1]. Effect of OS on expression of cytokines in the intestine has been investigated in previous studies, which can explain the direct effect of prebiotics on improving the immunity of the hosts [18]. Since the gut of newly hatched chicks is sterilized, the overall increased in the levels of immunoglobulins, particularly the IgG, in one-day old chicks administered *in ovo* OligoPKC is a clear evidence of the direct immunomodulatory effects of OligoPKC, including that of prenatal chicks, regardless of its prebiotic effect on intestinal microbiota and immunity of host due to the enhancement of beneficial microbiota. The beneficial immunomodulatory effect of *in ovo* OligoPKC on prenatal chicks may have some influence on the higher hatchability in the OligoPKC administered eggs. Although further enhancement in serum immunoglobulins production of neonatal chicks (up to 14 days old) were primarily influenced by dietary supplementation of OligoPKC post-hatching, *in ovo* OligoPKC injection seems to have only minor carry-over beneficial effect on the immune system in chicks as shown by the numerically higher immunoglobulins concentration in the PREBovo as compared to the control group.

Reported effects of OS on serum immunoglobulins production are not consistent. Kim et al. [19] showed that supplementation of fructo-OS and MOS had no effect on the levels of IgA, IgM and IgG in serum of broilers, while in another study, the concentration of the above-mentioned immunoglobulins significantly increased in pigs receiving galacto-mannan [20]. Other studies reported that dietary supplementation of MOS increased serum IgA in dogs [21] and laying hens [22], and increased IgG in layer hens [20]. Ito *et al*. [23] showed positive correlation between serum IgA concentration and the population of cecal *Lactobacillus* in rats receiving fructans, suggesting the indirect effects of prebiotics on immune system which is achieved through increasing the populations of beneficial bacteria. However, Roller [24] reported higher IgA secretion in the ileum of rats with supplementation of synbiotic (combination of probiotics and prebiotics) than supplementation of probiotics alone.

Prebiotic has been defined as non-digestible ingredients that increase the population of beneficial microorganisms [25] which leads to the reduction of the pathogenic microbes, such as *E*. *coli* or *Salmonella* in the gut of the host. The above indirect beneficial effect of prebiotics on the hosts is well documented in humans and animals. However, we know of only limited studies investigated the effect of *in ovo* injection of prebiotics on subsequent changes in the intestinal microbiota. Two previous studies [4,5] suggested that *in ovo* injection of prebiotics can modulate the population of intestinal microorganisms in post-hatch chickens. However, our result showed that dietary supplementation of OligoPKC was more effective than *in ovo* injection in beneficially altering the cecal microflora of the 14 days old chicks by increasing the population of beneficial bacteria and decreasing the population of pathogenic bacteria.

The decline in the expression of nutrient transporter genes in the *in ovo*-injected one-day old chicks and those fed OligoPKC at 14 days of age was rather surprising. However, the reduced genes expression did not negatively affected growth performance of the chickens, suggesting that nutrients intake was not affected. Although we did not collect data of the intestinal morphology to explain what could have happened, from published literature, we hypothesize that supplementation of MOS increased intestinal villi height and crypts depth resulting in better energy utilization [26,27,28] and thus, improved FCR. According to a study by Cheled-Shoval et al. [29], *in ovo* injection of MOS improved development of small intestine of chicks during pre- and post-hatch periods, including increased villi height, crypt depth and number of goblet cells per villus on the day of hatch in MOS treated birds.

High intestinal surface area, as a result of increased villi height and crypt depth, reduces the ratio of absorbable nutrients (peptides, fatty acids and carbohydrate molecules) to the number of epithelial cells (mainly enterocytes). Under this condition, since the expression of nutrient transporter genes has positive correlation with the level of available nutrient molecules, by increasing the surface area, the expression of these genes in each cell will be down-regulated.

It has been reported that carbohydrates and carbohydrate-containing biomolecules can be considered true antioxidants, capable of scavenging reactive oxygen species (ROS). Some studies showed that MOS has protection effect on heat-stressed chickens [30,31,32] and when supplemented to laying hens, significantly increased the total antioxidant activity of egg yolk and liver antioxidant enzymes [33]. Results of the present study are in agreement with the above researches, where chicks receiving OligoPKC showed high levels of antioxidant capacity in their liver and serum at both first and 14 days of age. Oskoueian *et al*. [34], reported that ethanolic extract of PKC contains high levels of fatty acids, phenolic compounds, sugar derivatives and other organic compounds with high antioxidant activity.

## Conclusion

Based on the results of the present study, although *in ovo* administration and dietary supplementation of OligoPKC did not affect the performance of broiler chicks, they, particularly dietary supplementation, improved the immune system of chicks by increasing the concentrations of serum immunoglobulins, enhanced their intestinal microbial balance of beneficial to pathogenic bacteria and increased the antioxidant capacity in serum and liver. Thus, OligoPKC is a potential prebiotic for chickens by enhancing effects on their health and well-being. OligoPKC down-regulated the expression of nutrient transporter genes; however, the down-regulation of these genes was not reflected in the FCR of the chicks and thus, this unexpected observation needs further investigation. Taking the above together, we conclude that *in ovo* injection enhances serum immunoglobulins particularly IgG production, antioxidant capacity in serum and liver during prenatal development in chicks, but had limited beneficial carrying-over effect on post-hatched chicks as compared to dietary supplementary of OligoPKC.

## Supporting information

S1 Appendix Experimental design. (DOCX)
