# Supplementary material for: In Ovo and dietary administration of oligosaccharides extracted from palm kernel cake influence general health of pre- and neonatal broiler chicks
Source: PLoS One. 2017 Sep 7;12(9):e0184553. doi: 10.1371/journal.pone.0184553 (PMC5589242; doi:10.1371/journal.pone.0184553)
Supplement: S1 Appendix — (DOCX) [file pone.0184553.s001.docx]

**Experimental design**
